# Characterization of Tigecycline-Heteroresistant *Klebsiella pneumoniae* Clinical Isolates From a Chinese Tertiary Care Teaching Hospital

**DOI:** 10.3389/fmicb.2021.671153

**Published:** 2021-08-03

**Authors:** Qiaoyu Zhang, Liping Lin, Yuhong Pan, Jiansen Chen

**Affiliations:** ^1^Department of Nosocomial Infection Control, Fujian Medical University Union Hospital, Fuzhou, China; ^2^Department of Laboratory Medicine, School of Medical Technology and Engineering, Fujian Medical University, Fuzhou, China; ^3^Department of Clinical Laboratory, Fujian Medical University Union Hospital, Fuzhou, China

**Keywords:** *Klebsiella pneumoniae*, tigecycline, heteroresistance, efflux pump, time-killing assay

## Abstract

Tigecycline has been used as one of the therapeutic choices for the treatment of infections caused by multidrug-resistant *Klebsiella pneumoniae*. However, the emergence of tigecycline heteroresistance has led to great challenges in treating these infections. The purpose of this study was to investigate whether tigecycline-heteroresistant *K. pneumoniae* (TGCHR-Kp) exists in clinical isolates, and to further characterize the underlying molecular mechanisms involved in the development of tigecycline-resistant subpopulations. Of the 268 tigecycline-susceptible clinical *K. pneumoniae* isolates, 69 isolates were selected as tigecycline-heteroresistant candidates in the preliminary heteroresistant phenotypic selection by a modified disk diffusion method, and only 21 strains were confirmed as TGCHR-Kp by the population analysis profile (PAP). Pulsed-field gel electrophoresis (PFGE) analysis demonstrated that all the parental TGCHR-Kp isolates were clonally unrelated, and colonies confirmed as the heteroresistant subpopulation showed no significant differences from their respective parental TGCHR-Kp isolates. Efflux pump inhibitors reversed the tigecycline susceptibility in heteroresistant subpopulations. Mutations in the *ramR* and *soxR* genes lead to upregulation of the *ramA* and *soxS* transcriptional regulators, which in turn induced overexpression of the AcrAB-TolC efflux pump genes in TGCHR-Kps-resistant subpopulations. Moreover, mutations of *rpsJ* were also found in resistant subpopulations, which suggested that the *rpsJ* mutation may also lead to tigecycline resistance. Time-kill assays showed that the efficacy of tigecycline against TGCHR-Kps was weakened, whereas the number of resistant subpopulations was enriched by the presence of tigecycline. Our findings imply that the presence of TGCHR-Kps in clinical strains causes severe challenges for tigecycline therapy in clinical practice.

## Introduction

*Klebsiella pneumoniae* (*K. pneumoniae*) is a critical nosocomial pathogen that can cause lower respiratory tract, urinary tract, lower biliary tract, and surgical wound site infections. With the emergence of multidrug-resistant (MDR) strains, carbapenems, and colistin have become alternative agents against MDR *K. pneumoniae* (Sader et al., 2015; Diep et al., 2018). However, the emergence of carbapenems and colistin resistance in *K. pneumoniae* has been a great challenge for clinicians (Doumith et al., 2009; Braykov et al., 2013).

Tigecycline is one of the few remaining therapeutic options for treating infections caused by carbapenem-resistant or MDR Gram-negative bacilli. Tigecycline belongs to a novel antibiotic class called glycylcyclines (Ahn et al., 2016), and it has an extensive antibacterial spectrum against most pathogens, including extended-spectrum β-lactamase-producing bacteria or carbapenemase-producing Gram-negative bacteria (Chen et al., 2012). Similar to tetracyclines, tigecycline reversibly binds to the ribosomal 30S subunit, interfering with bacterial amino acid translation and inhibiting bacterial growth (Olson et al., 2006). In comparison to tetracyclines, tigecycline can evade the classical tetracycline resistance mechanisms such as TetA- to TetE-mediated drug-efflux and ribosomal protection conferred by Tet(M) (Livermore, 2005). However, the prevalence of tigecycline-resistant *K. pneumoniae* is a cause for concern (Roy et al., 2013; Chiu et al., 2017). Previous studies (Horiyama et al., 2011; He et al., 2015) showed that among Enterobacteriaceae, decreased susceptibility to tigecycline was due to the AcrAB efflux pump undergoing upregulated expression (Horiyama et al., 2011; Veleba and Schneiders, 2012; Veleba et al., 2013; Li et al., 2019). The AcrAB-TolC efflux pump system has been widely investigated in *K. pneumoniae* (Ruzin et al., 2005; Roy et al., 2013; Xu et al., 2016). Expression of the *acrAB* genes is regulated by the local transcriptional repressor AcrR and the global transcriptional activators such as RamA, SoxS, and MarA (Schneiders et al., 2003; Bialek-Davenet et al., 2011; Rosenblum et al., 2011; Veleba et al., 2012; Wang et al., 2015; Blanco et al., 2016). The transcription of *marA* is direct repressed by MarR (Bialek-Davenet et al., 2011; He et al., 2015). As RamR directly represses the expression of *ramA*, mutations in *ramR* can cause the overexpression of *ramA* (Hentschke et al., 2010; He et al., 2015; Wang et al., 2015; Fang et al., 2016; Chiu et al., 2017). Besides, *soxS* induction depends on SoxR, and mutations within *soxR* can lead to its overexpression (Schneiders et al., 2003; Bialek-Davenet et al., 2011).

An RND family efflux pump, OqxAB has also been studied in MDR *K. pneumoniae* (Zhong et al., 2014). The expression of the OqxAB efflux pump genes is also controlled by the global activators RarA, MarA, and SoxS and the local repressor OqxR (Veleba and Schneiders, 2012; Veleba et al., 2012). Although tigecycline can evade some classical tetracycline efflux pumps Tet(A–E), tet(A) was thought to be responsible for tigecycline efflux (Foong et al., 2020). Overexpression and mutated tet(A) have been found to cause resistance of tigecycline, alone or in synergy with overexpression of the RND family efflux pump (Tuckman et al., 2000; Akiyama et al., 2013; Chiu et al., 2017; Foong et al., 2020). In addition, mutations in the *rpsJ* gene, which encodes the ribosomal cprotein S10, are associated with reduced tigecycline susceptibility (Beabout et al., 2015; Fang et al., 2016).

Tigecycline heteroresistance has already been found in some Gram-negative bacteria, such as *E. cloacae* and *Salmonella enterica serovar Typhimurium* (Chen et al., 2017; Liu et al., 2019). Heteroresistance is a phenomenon that describes the population-wide variable responses to antibiotics from bacterial populations occurring within the same progenitor cell (El-Halfawy and Valvano, 2013). Heteroresistance appears to be an intermediate stage for a bacterium transitioning from susceptible to resistant after exposure to certain antibiotic drugs (Falagas et al., 2008; Gefen et al., 2017), which may lead to the emergence of a resistant strain and ultimately contribute to antibiotic therapy failure (Moosavian et al., 2014; Gefen et al., 2017). However, the study of tigecycline heteroresistance in *K. pneumoniae* is rare.

## Materials and Methods

### Strains and Antibiotic Susceptibility Testing

Clinical *K. pneumoniae* strains were collected from patients with various clinical infections between August 2015 and October 2016 at Fujian Medical University Union Hospital, a tertiary-care teaching hospital with 2,500 beds located in southeastern China. The strains were stored at −80°C in Luria–Bertani (LB, Thermo Fisher Scientific, Shanghai, China) medium with 20% glycerol. In the following experiments, bacteria were reinoculated on MacConkey agar (Hope Bio, Shandong, China) for 24 h at 35°C, and a single colony from each agar plate was inoculated in 10 ml of liquid LB medium and incubated at 35°C for 24 h. A VITEK-2 Compact system (bioMérieux, Marcy l’Etoile, France) was applied to identify bacterial species and determine the minimum inhibitory concentration (MIC) for different antibiotics. The breakpoint for tigecycline was based on the criteria established by the European Committee on Antimicrobial Susceptibility Testing (EUCAST) guidelines, ≤1.0 mg/l as susceptible, 2.0 mg/l as intermediate, and ≥4.0 mg/l as resistant. The *K. pneumoniae* ATCC 13883 was used as a control strain.

### Selection of Tigecycline Heteroresistant Candidate Isolates

The disk diffusion method was adopted to screen tigecycline-heteroresistant candidate isolates by modifying methods of the previous study (Liu et al., 2019). Briefly, disks containing 15 μg tigecycline were placed on the Mueller–Hinton agar (MHA, Oxoid, Basingstoke, United Kingdom) plated with 0.5 McFarland standard fresh bacterial suspension. After incubation at 35°C for 24 h, any colony grown within the inhibition zone was selected as a candidate-heteroresistant subpopulation. The latter was identified with a VITEK-2 Compact instrument (bioMérieux, Marcy l’Etoile, France) to exclude any potential contamination.

### Population Analysis Profiling and Passage Stability

Population analysis profiling (PAP) was conducted to confirm the heteroresistance phenotype as previously described (Meletis et al., 2011). Briefly, 50-μl aliquots of overnight culture were suspended in 5 ml of LB broth and grown to the mid-logarithmic phase (OD_6__00_ = 0.3∼0.4). The bacterial pellet was collected by centrifugation and resuspended in MH broth (Oxoid, Basingstoke, United Kingdom). Serially diluted bacterial suspensions from 10^8^ to 10^2^ CFU were prepared and spread on MH agar plates (Oxoid, Basingstoke, United Kingdom) containing 0.5, 1, 2, 4, 8, 16, and 32 mg/l tigecycline. Following 48 h incubation at 35°C, colonies that grew on the plates were counted. The frequency of heteroresistant subpopulations was calculated by dividing the number of colonies grown on the highest drug plate by the number of colonies from the same inoculum of the antibiotic-free plate (Meletis et al., 2011). The experiments were repeated three times, and the mean of viable CFU was plotted on a semilogarithmic graph. The detection limit of tigecycline-resistant subpopulations was 20 CFU/ml. Tigecycline-heteroresistant *K. pneumoniae* (TGCHR-Kp) was defined as the presence of a subpopulation of cells capable of growing at a concentration of drugs at least twofold higher than those of tigecycline-susceptible parental strains. Subpopulations grown on the highest concentration of tigecycline plates were randomly picked up and serially passaged daily on antibiotic-free medium for 14 days. The MICs were reassessed by the broth microdilution (BMD) method to evaluate whether the resistant phenotypes were stable (Meletis et al., 2011; Liu et al., 2019).

### Pulsed-Field Gel Electrophoresis

Pulsed-field gel electrophoresis (PFGE) was performed for all of the TGCHR-Kps strains as described in the PulseNet protocol (Ribot et al., 2006). All chemicals, unless stated otherwise, were purchased from Sigma Chemical Co. (Sigma, St. Louis, MO, United States). Briefly, cells cultured on blood agar for 18 h were adjusted to an OD610 of 1.3–1.4 in the cell suspension buffer (CSB, 100 mM Tris–HCl: 100 mM EDTA, pH 8.0) and resuspended in 400 μl CSB with 20 μl of proteinase K (20 mg/ml). The cell suspension was mixed with an equal volume of 1.0% (w/v) SeaKem Gold Agarose (Lonza, Rockland, ME, United States) to form the plugs. These plugs were incubated in 2 ml of Cell Lysis Buffer (50 mM Tris–HCl: 50 mM EDTA, pH 8.0, with 1% *N*-lauroyl-sarcosine, sodium salt (Sarcosyl), 0.1 mg/ml Proteinase K) at 54°C for 2 h. The plugs were washed five times at 50°C for 15 min with sterile water and were digested at 37°C for 2 h with 50 U of *Xba*I (Thermo Scientific, Vilnius, Lithuania). Then, the plugs were placed on the well comb and 20 ml preheated agarose was poured into the gel mold. The gel was run with a CHEF Mapper system (Bio-Rad, Hemel Hempstead, United Kingdom) at 6.0 V/cm with an initial switch time of 2.16 s to a final switch time of 54.17 s in 0.5× Tris-borate-EDTA running buffer at 14°C for 18 h. The DNA band profiles were stained with ethidium bromide and visualized and digitized by a dendrogram which was developed using Bionumerics analysis software (v7.6) (Applied Maths, Belgium). Percent similarities were described by the unweighted-pair group method using arithmetic averages (UPGMA), which was based on the Dice coefficient according to the different positions and numbers of bands by electrophoresis.

### Evaluation of Bacteria Efflux Pump Activity

Two types of efflux pump inhibitors, phenylalanine-arginine β-naphthylamide (PAβN, Sigma, St. Louis, MO, United States) and carbonyl cyanide 3-chlorophenylhydrazone (CCCP, Sigma, St. Louis, MO, United States), were used to evaluate the efflux pump activities by determining the MICs for both the parental strains and the subpopulations (Zhong et al., 2014; Liu et al., 2019). Briefly, twofold serial tigecycline-containing agar plates were prepared in the presence or absence of 20 mg/l PAβN and 25 mg/l CCCP. Test isolates were grown overnight to mid-logarithmic phases (OD_600_ = 0.3–0.4), and the pellets were harvested and resuspended in LB broth at a concentration of approximately 5 × 10^6^ CFU/ml. Two microliters aliquots were pipetted on the MHA surface to achieve a final inoculum of approximately 10^4^ CFU per spot and then incubated at 35°C for 24 h. Both the parental strains and the subpopulations were tested in triplicate.

### Time-Killing Assay

Time-killing assays were conducted on five randomly selected strains, by inoculating approximately 10^6^ CFU/ml of the culture into cation-adjusted Mueller–Hinton broth (CAMHB) containing 4 mg/l tigecycline. The cultures were incubated with shaking (37°C, 200 rpm) and 50-μl aliquots were removed, serially diluted 10-fold in PBS, and plated on plates at the defined timepoints (0, 2, 4, 6, 8, 10, 24, and 48 h after inoculation) to count the CFU. Serially diluted cultures were also plated on CAMHB with 4 mg/l tigecycline to select the resistant colony. The limit of tigecycline-resistant subpopulations was 20 CFU/ml. All experiments were performed in triplicate.

### Quantitation of the Regulator Genes and the Efflux Pumps by Real-Time PCR

The expression levels of the regulator gene *ramA*, *marA*, *soxS*, and *acrR*, and the efflux components OqxAB and AcrAB-TolC for the parental strains and their respective resistant subpopulations were assessed by quantitative real-time PCR (qRT-PCR). Bacteria grown to mid-logarithmic phase and total RNA were extracted by using TRIzol reagent (Tiangen, Beijing, China). Total RNA was digested with DNase I to remove a potential contamination of genomic DNA. The yield and quality of RNA were measured by using a NanoDrop 2000C photometer (Thermo, Chicago, IL, United States). The cDNA was synthesized from 600 ng of total RNA using an Eastep RT Master Mix Kit (Promega, Shanghai, China). The primers for *acrA*, *acrB*, *tolC*, *ramA*, *marA*, *soxS*, *oqxA*, *oqxB*, and *rrsE* are listed in the Supplementary Table 1. Real-time PCR was performed with a Roche Light Cycler SYBR Green I Master kit on a LightCycler 480 instrument (Roche, Branchburg, NJ, United States) in triplicate. PCR was carried out for 2 min at 95°C for initial denaturation, and 45 cycles of 5 s at 95°C, 30 s at 55°C, and 30 s at 72°C. Fluorescence was collected at every cycle of the extension period. Melting curve analysis was conducted from 55 to 95°C with a rate of 0.02°C/s increment. The 2^–ΔΔCT^ method was used to calculate the relative expression of genes by normalizing it to the *rrsE* housekeeping gene. The relative expression of each target gene for the parental strains and the subpopulations was then compared with that for the *K. pneumoniae* ATCC 13883.

### PCR Amplification and Sequencing

Whole-cell DNA was extracted by the boiling method, and the *acrR*, *ramR*, *ramA*, *soxR*, *soxS*, *marR*, *marA*, *tetA*, and *rpsJ* genes were amplified with the primers listed in the Supplementary Table 1. The amplified DNA fragments were purified and sequenced with an ABI 3730 sequencer (Applied Biosystems, Foster City, CA, United States). The nucleotides and deduced protein sequences were analyzed by the DNAMAN software version 8.0 (Lynnon Biosoft, United States) and compared with the reference sequence of *K. pneumoniae* MGH78578 (accession number: NC_009648; CP000647). The sequences were submitted to GenBank, and their corresponding accession numbers are presented in Table 3.

**TABLE 1:** Characterization of the TGCHR-Kps phenotypes.

**Strains**	**Parental strain MIC (mg/l)**	**Highest concentration of growth in PAP (mg/l)**	**Frequency of resistant subpopulations**	**Resistant subpopulations MIC (mg/l)**	**Resistant subpopulations MIC after 14-day passages (mg/l)**
K10	1	8	1.04 × 10^–6^	8	8
K24	0.5	2	2.67 × 10^–6^	4	4
K26	0.5	4	1.82 × 10^–5^	4	4
K86	0.5	4	9.80 × 10^–7^	4	4
K89	0.5	4	3.1 × 10^–6^	4	4
K98	1	4	1.13 × 10^–5^	4	4
K116	0.5	4	1.14 × 10^–6^	4	4
K118	0.5	2	5.52 × 10^–6^	4	4
K130	0.5	4	1.33 × 10^–6^	4	4
K148	1	4	1.04 × 10^–5^	4	4
K151	0.5	4	4.36 × 10^–6^	4	4
K182	0.5	4	1.82 × 10^–6^	4	4
K191	0.5	4	2.32 × 10^–6^	4	4
K194	0.5	2	1.32 × 10^–6^	4	4
K197	0.5	2	6.62 × 10^–6^	4	4
K228	1	4	2.97 × 10^–6^	4	2
K289	0.5	4	5.92 × 10^–7^	4	2
K295	1	4	4.57 × 10^–7^	4	4
K300	0.5	4	3.54 × 10^–6^	4	4
K320	1	4	1.33 × 10^–5^	4	4
K326	0.5	16	1.41 × 10^–6^	16	8

**TABLE 2:** Tigecycline MICs with or without efflux pump inhibitors in parental and unsusceptible subpopulations of TGCHR-Kps.

**Isolates**	**Parental strains MIC (mg/l)**			**Resistant subpopulations MIC (mg/l)**		
	**TGC**	+PAβN*	**+CCCP^#^**	**TGC**	+PAβN*	**+CCCP^#^**
K10	1	1	1	8	2	4
K24	0.5	0.5	0.5	4	0.5	0.5
K26	0.5	0.5	0.5	4	0.5	2
K86	0.5	0.5	0.5	4	0.5	0.5
K89	0.5	0.5	0.5	4	0.5	2
K98	1	0.5	0.5	4	0.5	1
K116	0.5	0.5	0.5	4	1	2
K118	0.5	0.5	0.5	4	0.5	0.5
K130	0.5	0.5	0.5	4	1	2
K148	1	0.5	0.5	4	0.5	2
K151	0.5	0.5	0.5	4	1	1
K182	0.5	0.5	0.5	4	1	1
K191	0.5	0.5	0.5	4	1	1
K194	0.5	0.5	0.5	4	0.5	0.5
K197	0.5	0.5	0.5	4	1	1
K228	1	1	0.5	4	0.5	1
K289	0.5	0.5	0.5	4	0.5	1
K295	1	0.5	0.5	4	0.5	1
K300	0.5	0.5	0.5	4	0.5	2
K320	1	0.5	0.5	4	0.5	2
K326	0.5	0.5	0.5	16	4	8
ATCC 13883	0.5	0.5	0.5	NA	NA	NA

*The MHA containing 20 mg/l PAβN.

*^#^The MHA containing 25 mg/l CCCP. ATCC 13883 was used as a control strain.*

**TABLE 3:** Mutations of *ramR*, *soxR*, *marR*, *acR*, and *rpsJ* genes in unsusceptible subpopulations.

**Isolates**	**ramR**	**soxR**	**marR**	**acrR**	**rpsJ**
K10	p. H186N (KY933482)	p.D142E (MH838030)	Wild	p.V165I (MZ126594)	g.C11ins (MH838021)
K24	p. A166S (KY933483)	p.A15S, D142E (MH838032)	Wild	Wild	Wild
K26	g.77-78DelAT (KY933484)	p.A15S (MH838033)	Wild	Wild	Wild
K86	Wild	p.D142E (MH838031)	Wild	Wild	g.C11ins (MH838022)
K89	p. G42E (KY933485)	Wild	Wild	p.R22S (MZ126595)	Wild
K98	p. A 19V (KY933486)	Wild	Wild	Wild	Wild
K116	Wild	Wild	Wild	Wild	g.C11ins (MH838023)
K118	p. H186N(KY933487)	Wild	Wild	p.V165I p.Y114F (MZ126596)	g.C305ins (MH838024)
K130	Wild	Wild	Wild	Wild	g.C11ins (MH838025)
				
K148	p. Q122* (KY933489)	Wild	Wild	p.R90G (MZ126597)	Wild
K151	p. H186N (MF324919)	Wild	Wild	p.Y114F p.V165I (MZ126598)	g.C11ins, C305ins (MH838026)
K182	g.515 ins C (KY933490)	D142E (MH838034)	Wild	g.T75ins (MZ126599)	Wild
K191	g.571DelG (KY933491)	Wild	Wild	Wild	Wild
K194	p. G25C; p. H186N (KY933492)	p.D142E (MH838035)	Wild	p.Y114F p.V165I (MZ126600)	Wild
K197	g.124-125ins AG (KY933494)	Wild	Wild	Wild	Wild
K228	Wild	Wild	Wild	Wild	g.C302ins (MH838027)
K289	Wild	Wild	Wild	Wild	g.C302ins (MH838028)
K295	Wild	Wild	Wild	Wild	g.CC11-12ins, C305ins (MH838029)
K300	p. F48S (KY933495)	Wild	Wild	g. 281ins ISKpn26 (MZ126601)	Wild
K320	g.Del(379–386) (MF324922)	Wild	Wild	Wild	Wild
K326	g:Del45bp (537 ∼ 582) (KY933501)	Wild	Wild	Wild	Wild

*The sequence accession numbers of gene mutation are listed in parentheses.*

### Statistical Analysis

Statistical analysis was performed by the Prism 8.4.0 (GraphPad Software) software. The expression levels of *acrA*, *acrB*, *tolC*, *oqxA*, *oqxB*, *marA*, *ramA*, *acrR*, and *soxS* between the parental strains and the subpopulations were determined by two-tailed Student’s *t*-test. A *p*-value less than 0.05 was considered statistically significant.

## Results

### Determination of TGCHR-Kps

**FIGURE 1:**
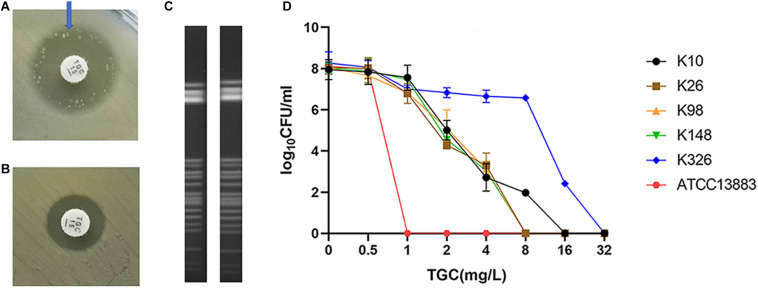
Screen and confirmation of TGCHR-Kps. **(A)** The colonies scattered within the TGC-containing disk inhibition zone (21 mm in diameter) of a K148 clinical isolate. **(B)** The arrow indicates bacterial colony disk inhibition zone (12 mm in diameter) determined by a *Kirby–Bauer* method. **(C)** The homology between a TGCHR-Kp(K148) and its homologous resistant subpopulation was analyzed by PFGE. **(D)** PAP was carried out in triple repeats to confirm TGCHR-Kps in five clinical isolates; ATCC 13883 was used as the control strain.

**FIGURE 2:**
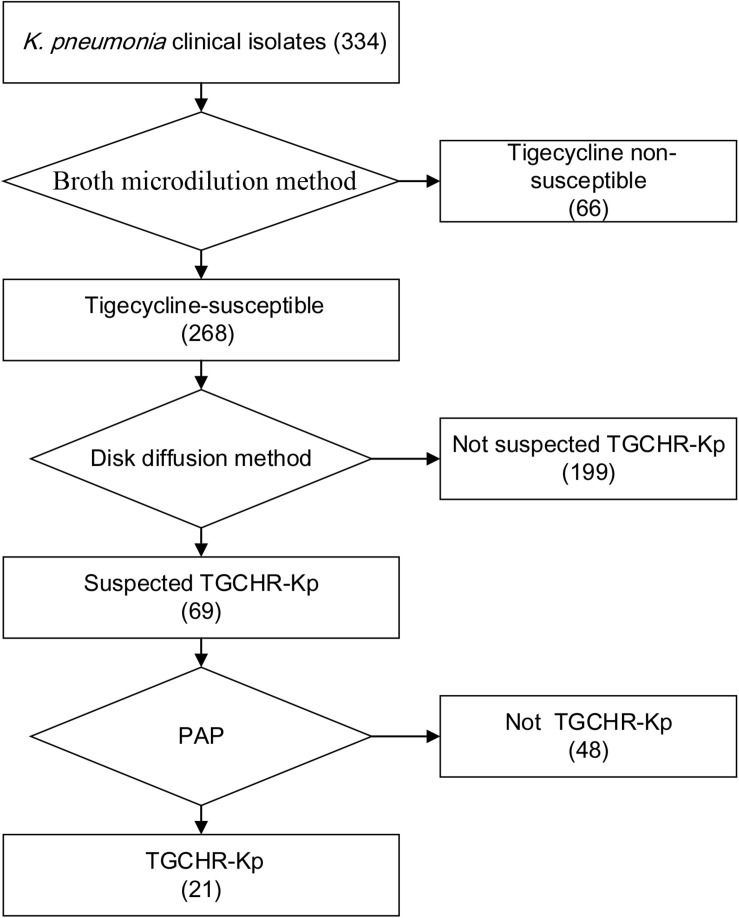
Flowchart of TGCHR-Kp screening and confirmation. The broth micro-dilution method was used to determinate the tigecycline MIC values. The disk diffusion method was applied for selecting the TGCHR-Kp candidates. Moreover, the population analysis profiling (PAP) was conducted to confirm the TGCHR-Kp isolates. Numbers in braces indicate the number of strains.

Of the 334 clinical *K. pneumoniae* strains, 268 isolates were susceptible to tigecycline, which was detected by the automated BMD method. Then, in the preliminary selection of candidate tigecycline-heteroresistant strains measured by the modified disk diffusion method, more than a quarter of the tigecycline-susceptible isolates (69/268) presented tiny and sporadic colonies in the clear zone of inhibition (Figure 1A). The subpopulation showed less susceptibility than its respective parental strain (Figure 1B). The inhibition zone diameters of parental strains and respective resistant subpopulations are shown in the Supplementary Table 2. In addition, the parental strain and its respective resistant subpopulation showed a similar PFGE band pattern (Figure 1C), indicating that they were isogenic. PAP assays confirmed that the resistant subpopulations of the five tested heteroresistant strains could grow in tigecycline concentrations as high as 16 mg/l (Figure 1D). Only 21 isolates were confirmed as TGCHR-Kps by the PAP gold standard method. The flowchart of selection and confirmation for TGCHR-Kp is shown in Figure 2. The subpopulations grown in the highest tigecycline concentration had a 4- to 32-fold MIC increment (ranging from 4 to 16 mg/l), when compared with their parental strains, respectively. The proportion of resistant subpopulations ranged from 4.57 × 10^–7^ to 1.82 × 10^–5^ (Table 1).

### Homologies Among TGCHR-Kps

**FIGURE 3:**
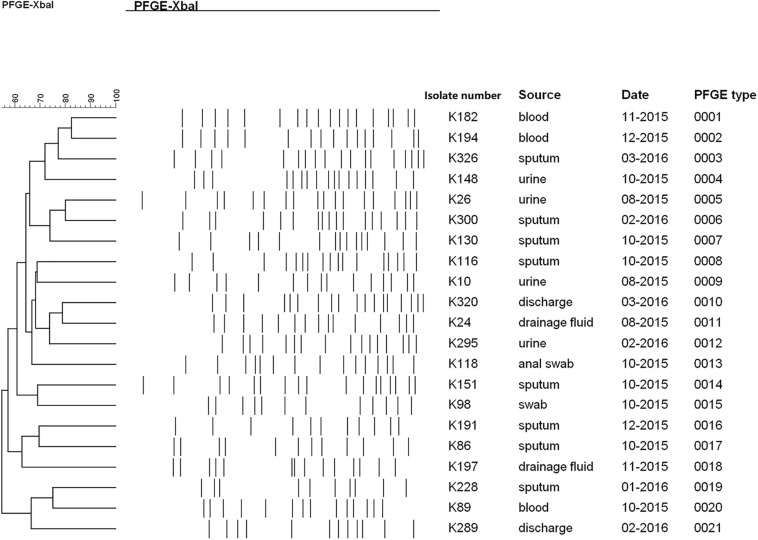
Homology analysis of TGCHR-Kps. Homology of TGCHR-Kps was identified by PFGE. The dendrogram was developed by using Bionumerics analysis software. Percent similarities are described by unweighted-pair group method using arithmetic averages (UPGMA).

PFGE was performed to identify the homologies among the TGCHR-Kp clinical isolates and the homologous subpopulations. All TGCHR-Kps were classified into distinct patterns, indicating that TGCHR-Kps were epidemiologically unrelated (Figure 3). Additionally, both the parental strains and their respective subpopulations displayed similar PFGE band patterns, further confirming the presence of heteroresistance among clinical isolates (Figure 1C).

### Stability of Tigecycline Unsusceptible Subpopulations

To determine whether the tigecycline-unsusceptible subpopulations were stable, the subpopulations were serially passaged on tigecycline-free medium for 14 days, and then tigecycline susceptibility was evaluated. After subculture onto a tigecycline-free medium, 18 subpopulations retained the same MIC values, whereas 3 subpopulations had a twofold decrease in tigecycline MICs. All the 21 subpopulations’ MIC retained the unsusceptible range (Table 1).

### Efflux Pump Inhibitors Restore Tigecycline Susceptibility

To assess whether the efflux pumps were responsible for reduced tigecycline susceptibility in the TGCHR-Kp subpopulations, two efflux pump inhibitors, PAβN and CCCP, were used to evaluate the efflux activity. Each of the subpopulations had a two- to eightfold decrease in tigecycline MIC in the presence of either PAβN or CCCP, indicating the role of efflux pumps in decreasing the tigecycline susceptibility (Table 2).

### Overexpression of the AcrAB-TolC Efflux Pump

**FIGURE 4:**
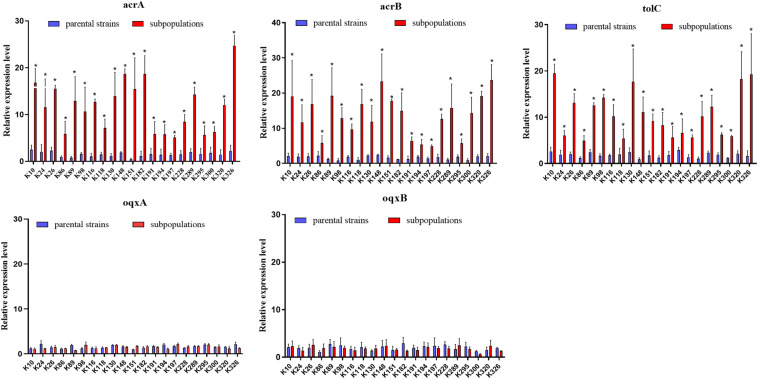
Expression of the AcrAB-TolC and OqxAB efflux pump genes in TGCHR-Kps. The relative expressions of *acrA*, *acrB*, *tolC*, *oqxA*, and *oqxB* genes between parental strains and subpopulations were measured by real-time PCR and normalized to those of the rrsE housekeeping gene by the 2^–ΔΔCT^ method. The experiment was repeated three times. Mean relative expression (delta CT values) and standard deviation (SD) are shown. The statistically significant difference is represented by * means *p* < 0.05.

**FIGURE 5:**
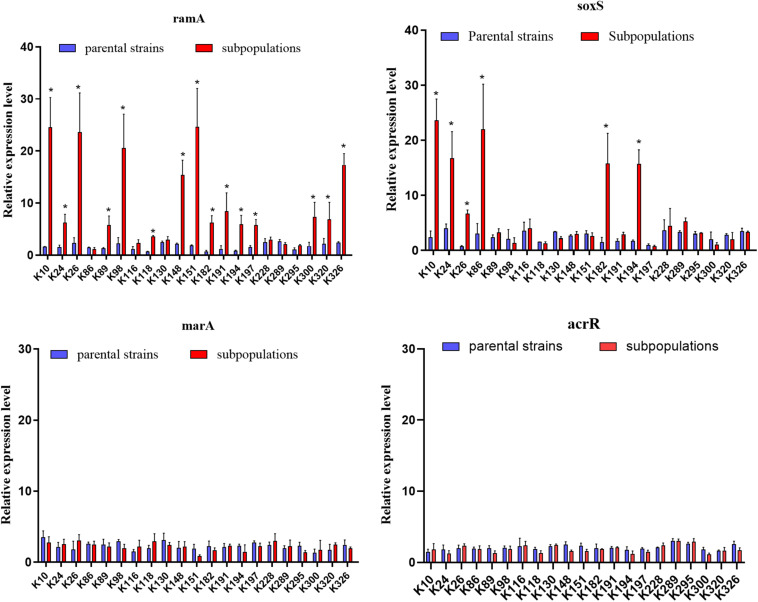
Expression of global regulators *ramA*, *soxS*, *marA*, and local regulator *acrR* genes. The relative expressions of *ramA*, *soxS*, *marA*, and *acrR* genes between parental strains and resistant subpopulations were measured by real-time PCR and normalized to those of the rrsE housekeeping gene by the 2^–ΔΔ*CT*^ method. The experiment was repeated in triplicate. The mean relative expression levels (delta CT values) and standard deviation (SD) are shown. The statistically significant difference is represented by * mean *p* < 0.05.

We further evaluated the expression levels of efflux pumps AcrAB-TolC (AcrA, AcrB, and TolC) and the role of regulators (RamA, MarA, and SoxS) in the form of resistant subpopulations in the TGCHR-Kp. In comparison with their parental strains, qRT-PCR analysis showed significant upregulation of AcrA (3.46- to 35.93-fold) in all subpopulations, as well as AcrB (2.7- to 16.25-fold) and TolC (2.56- to 11.99-fold) (Figure 4). For these subpopulations, overexpression of regulator RamA (3.68- to 14.16-fold) was detected in 71.4% (15/21) of the subpopulations and overexpression of SoxS (4.17- to 10.08-fold) was detected in 28.6% (6/21) of the subpopulations, while none of the subpopulations overexpressed MarA (Figure 5). We also evaluated the expression levels of the efflux pumps OqxAB (OqxA and OqxB). However, we did not find *oqxAB* overexpression in the resistant subpopulations (Figure 4).

### Mutations in Regulators Confer to the Overexpression of Global Transcriptional Activators

We finally investigated mutations of the *acrR*, *ramR*, *soxR*, *marR*, *tetA*, and *rpsJ* genes in TGCHR-Kp. Among the tigecycline-resistant subpopulations, eight isolates harbored mutations within the *acrR* genes, and 15 isolates harbored mutations in the *ramR* gene that caused higher expression of *ramA* (3.68- to 14.16-fold) than parental strains. Six subpopulations harbored mutations of the *soxR* gene resulting in increased expression of *soxS* (4.17- to 10.08-fold), respectively. No mutations were found in the *marR* gene. Accordingly, no *marA* overexpression was detected. In addition, nine subpopulations harbored mutations in the *rpsJ* gene. However, we could not find tetA-positive-resistant subpopulations (Table 3).

### *In vitro* Effect of Tigecycline Against TGCHR-Kps

**FIGURE 6:**
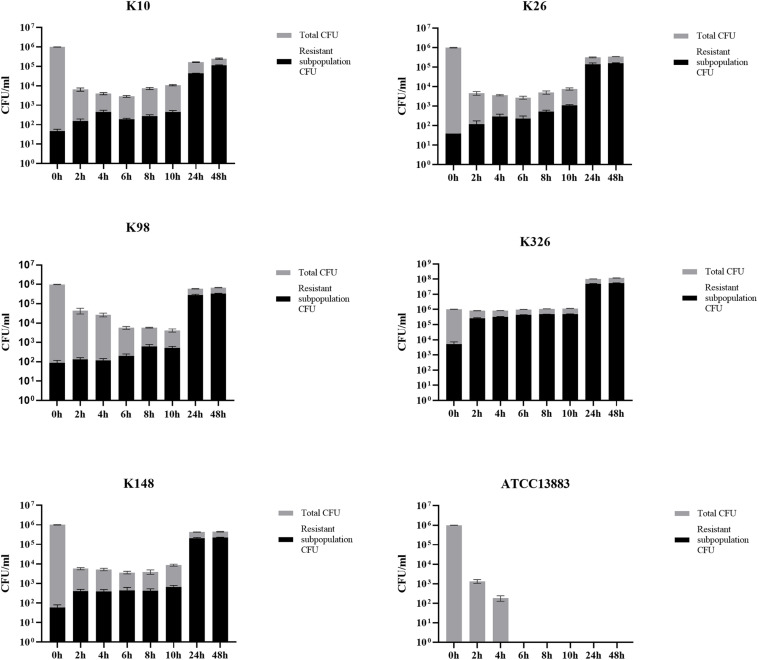
The proportion of TGC-resistant subpopulations during time-killing analysis. The TGCHR-Kps cultures were treated with 4 mg/l TGC for 48 h. Bacteria were plated at the indicated timepoints for counting the total CFU (gray column) and the homologous resistant (black column) CFU. Error bars indicate standard deviations of triple repeated experiments. ATCC 13883 was used as a control strain.

In the time-killing assay, the control isolate ATCC 13883 was quickly eliminated by 4 mg/l tigecycline and could not detect any bacteria at the end of 6 h (Figure 6), while all tigecycline-treated heteroresistant isolates only led to *a* ≤ 3 log10 reduction at the first 10 h and then significantly resumed growth after an extended incubation (≥24 h) (Figure 6). We then assessed whether the resistant subpopulations were specifically enriched in the presence of tigecycline. It is evident that the frequency of resistant subpopulations increased under tigecycline pressure. The resistant subpopulations gradually became predominant after the later incubation period (Figure 6). It is implied that the presence of heteroresistant subpopulations could lead to the emergence of TGC-resistant strains and treatment failure.

## Discussion

In this study, we first report the presence of a tigecycline-heteroresistant phenotype among *K. pneumoniae* clinical isolates. The overexpression of the AcrAB-TolC efflux pump and mutations of the *rpsJ* gene might contribute to the reduced susceptibility to tigecycline in the resistant subpopulations. The weakening effect of tigecycline against the TGCHR-Kps was due to the enrichment of resistant subpopulations during tigecycline treatment *in vitro*. Our findings demonstrate that the presence of TGCHR-Kps may pose a severe challenge to tigecycline therapy in clinical practice.

Heteroresistance has been found in *K. pneumoniae* for carbapenems or colistin, *Pseudomonas aeruginosa* for carbapenems, and *Acinetobacter baumannii* for colistin (Pournaras et al., 2007; Meletis et al., 2011; Moosavian et al., 2014; Adams-Sapper et al., 2015). In TGCHR-Kps, tigecycline-resistant subpopulations occurred at relatively low frequencies (4.57 × 10^–7^ to 1.82 × 10^–5^), indicating that such subpopulations would enter the susceptible range by the conventional BMD method. Low-frequency heteroresistant bacterial subpopulations can contribute to clinically relevant antibiotic resistance. In addition, the resistant subpopulations are metastable, implying that heteroresistant subpopulations might play an important role in the development of bacterial resistance (Pournaras et al., 2007; Meletis et al., 2011). Therefore, our results imply that antibiotic therapy not considering resistant subpopulations will lead to an increased frequency of its resistant subpopulations and cause the therapy to fail, which agrees with previous studies on the *in vitro* effect of antibiotics treating heteroresistant strains (Moosavian et al., 2014; Ma et al., 2019).

Many regulatory mechanisms of the AcrAB-TolC efflux pump have been described in other clinically relevant bacterial species. In *K. pneumoniae*, tigecycline resistance is mainly associated with the overexpression of efflux pumps, especially AcrAB-TolC and OqxAB (Ruzin et al., 2005, 2008; Kallman et al., 2008). The AcrAB-TolC efflux pump systems in the Enterobacteriaceae family are regulated by the local regulator AcrR and the global regulators MarA, SoxS, and RamA (Adler et al., 2016). Among the TGCHR-Kp-resistant subpopulations, nine resistant subpopulations harbored mutations in the *acrR* gene. Of note, one isolate (K300R) has an integration of ISKpn26 into the *acrR* gene, leading to the deactivation of *acrR* and increased expression of *acrAB*. While K300 is a KPC-producing *K. pneumoniae*, Yang et al. (2021) reported a strong link between KPC-2 plasmid-located ISKpn26 and ISKpn26 insertion into *acrR*, indicating that the blaKPC-2 plasmid is the reservoir for ISKpn26. However, we could not find a lower expression of *acrR* in the corresponding resistant subpopulation. Schneiders et al. (2003) report that an increased level of transcription of *acrA* follows increased levels of expression of regulatory genes *marA* and *soxS*, even in the presence of a functioning AcrR. The majority of subpopulations (15/21) harbored various types of mutations within the *ramR* gene, including nucleotide substitution, insertion, or deletion. Mutations within the *ramR* gene were found in both the DNA- and ligand-binding domains of the protein and were involved in overexpression of the *ramA* gene, which upregulated AcrAB-TolC efflux pump production, supporting the role of RamA in reduced tigecycline susceptibility, which is consistent with a previous study (Schneiders et al., 2003; Rosenblum et al., 2011; De Majumdar et al., 2015). In addition, six subpopulations exhibited mutations within the *soxR* gene, which resulted in the *soxS* overexpression and upregulated AcrAB-TolC efflux pump expression (Bialek-Davenet et al., 2011). MarA might serve as an alternative regulator, which could also affect the *acrB* efflux pump gene and, consequently, contribute to tigecycline and even cross-drug resistance (Zhong et al., 2014; Park et al., 2020). Nevertheless, in contrast with previous reports (Bratu et al., 2009; Veleba et al., 2012; Xu et al., 2016), there were no mutations harbored in the marR gene, and the global regulator MarA did not show an obvious overexpression in the subpopulations. The expression of the *oqxAB* efflux pump genes is also regulated by the global activators RarA, MarA, and SoxS and the local repressor OqxR (Park et al., 2020). There was no increase in the expression level of the *oqxAB* in the TGCHR-Kps, which is inconsistent with previous studies (Zhong et al., 2014; Chen et al., 2017; Park et al., 2020). Efflux pump TetA can be associated with mobile genetic elements such as transposons and plasmids, contributing to a rapid spreading of this mechanism (Akiyama et al., 2013; Xu et al., 2021). Thus, surveillance for the prevalence and dissemination of *tetA* is important to decrease tigecycline resistance in pathogenic bacteria. However, the absence of *tetA* in both parents and subpopulations of *K. pneumoniae* in our study suggests that tigecycline heteroresistance might not result from the TetA efflux pump.

While efflux pumps are major contributors to TGC resistance, mutations in *rpsJ*, the gene that encodes the ribosomal S10 protein, a component of the 30S ribosomal subunit and participant in the formation of a BoxA-binding module, may also confer reduced susceptibility to TGC in both Gram-positive and Gram-negative pathogens, indicating that mutations in *rpsJ* may provide a general target for reduced TGC susceptibility (Gallegos et al., 1997; Beabout et al., 2015). The V57 L mutation leading to tigecycline resistance has been found in previous studies (Villa et al., 2014; Fang et al., 2016; He et al., 2018). In this study, only nucleic acid insertion of the *rpsJ* gene was found in nine subpopulations. The insertion positions occurring at 11, 302, and 305 are common. Nucleotide insertions result in frameshifts and form truncated proteins, which might weaken the tigecycline banding to 16S rRNA, leading to tigecycline resistance.

Notably, more than one mechanism may contribute to tigecycline resistance in the subpopulations. Five resistant subpopulations (K116, K130, K228, K289, and K295) showed no mutations within the *acrR*, *ramR*, *soxR*, and *marR* regulator genes but showed overexpression of the *acrAB* efflux pump genes, which implies that other genetic mechanisms might be involved in the overexpression of the *acrAB* efflux pump in *K. pneumoniae*. Moreover, further study will be required to determine whether the efflux pumps or mutations within the *rpsJ* gene play unique roles in the development of a heteroresistance phenotype in *K. pneumoniae*.

The limitation of this study is that not all the TGCHR-Kps were MDR clinical strains. Our future study will be focused on determining tigecycline heteroresistance in carbapenemase-resistant *K. pneumoniae*. However, it does provide some clues on the prevalence of TGCHR-Kp and addresses the mechanism of tigecycline resistance in TGCHR-Kp. There is an urgent need to explore new treatment options for the TGCHR-Kp strains.

## Data Availability Statement

The datasets presented in this study can be found in online repositories. The names of the repository/repositories and accession number(s) can be found in the article/Supplementary Material.

## Author Contributions

YP collected the strains and performed the broth microdilution (BMD) method to determine tigecycline MICs. LL performed PFGE and PCR. QZ performed the selection of TGCHR-Kps, efflux activity assessment, and quantitative real-time PCR and was a major contributor in writing the manuscript. JC analyzed the data and gave advice for the experiments. All the authors read and approved the final manuscript.

## Conflict of Interest

The authors declare that the research was conducted in the absence of any commercial or financial relationships that could be construed as a potential conflict of interest.

## Publisher’s Note

All claims expressed in this article are solely those of the authors and do not necessarily represent those of their affiliated organizations, or those of the publisher, the editors and the reviewers. Any product that may be evaluated in this article, or claim that may be made by its manufacturer, is not guaranteed or endorsed by the publisher.
