# Supplementary material for: Characterization of Tigecycline-Heteroresistant Klebsiella pneumoniae Clinical Isolates From a Chinese Tertiary Care Teaching Hospital
Source: Front Microbiol. 2021 Aug 3;12:671153. doi: 10.3389/fmicb.2021.671153 (PMC8369762; doi:10.3389/fmicb.2021.671153)
Supplement: Supplementary file 1 [file Table_1.DOCX]

Supplementary Material

**Table S1** List of primers used in experiments

| Primers | Sequence(5’-3’) | References |
| --- | --- | --- |
| Real-time PCR | | |
| *acrA* F | ATGTGACGATAAACCGGCTC | Kallman et al., 2008 |
| *acrA* R | CTGGCAGTTCGGTGGTTATT |  |
| *acrB* F | CGATAACCTGATGTACATGTCC | Doumith et al.,2009 |
| *acrB* R | CCGACAACCATCAGGAAGCT |  |
| *tolC* F | CTACAAACAGGCGGTGGTCT | Roy et al., 2013 |
| *tolC* R | TGTTCAGCTCGTTGATCAGG |  |
| *soxS* F | GCATCACGGTACGGAACAT | Bratu et al., 2009 |
| *soxS* R | AGTCGCCAGAAAGTCAGGAT |  |
| *ramA* F | GCATCAACCGCTGCGTATT | Ruzin et al.,2005 |
| *ramA* R | CGTTGCAGATGCCATTTCG |  |
| *oqxA* F | TATCGCGCCACGCTGGAACAGG | Liu et al., 2019 |
| *oqxA* R | CGGCGCTGCTCCCACTCTTC |  |
| *oqxB* F | GATCAGGCGCAGGTTCAGGTGCA | Liu et al., 2019 |
| *oqxB* R | AACAGATGCACCACCAGCGTCAG |  |
| *rrsE* F | TTGACGTTACCCGCAGAAGAA | Ruzin et al.,2005 |
| *rrsE* R | GCTTGCACCCTCCGTATTACC |  |
| *acrR* F | CTACGTCTTTTCTCACAGC | Adler et al., 2016 |
| *acrR* R | CCCAGATCTCACTGAACAA |  |
| Sequencing |  |  |
| *ramR* F | CACGGTTCATATCCTGACCA | Bialek-Davenet et al., 2011 |
| *ramR* R | CCRTCGACCTTAAACACGTC |  |
| *marA-marR* F | CATAGCTGAGGCTGGAGRCC | Bialek-Davenet et al., 2011 |
| *marA-marR* R | TCGGCCAATTCATAATGTTG |  |
| *soxS-soxR* F | CGGAACCTCCATCAACAGATT | Bialek-Davenet et al., 2011 |
| *soxS-soxR* R | GCAGGTAAGCTGGCTCTACAA |  |
| *rpsJ* F | GGATCCCAATCGTAATGGGTATGAGGAG | Fang et al., 2016 |
| *rpsJ* R | GGATCCTAACACGGTTTGCTTCAACTT |  |
| *acrR* F | CGTAACCTCTGTAAAGTCAT | Kallman et al., 2008 |
| *acrR* R | GCTGACAAGCTCTCCGGGC |  |
| *tetA F* | GCCTTTCCTTTGGGTTCTCT | Akiyama et al., 2013 |
| *tetA R* | TGTCCGACAAGTTGCATGAT |  |
