# Supplementary material for: Characterization of Tigecycline-Heteroresistant Klebsiella pneumoniae Clinical Isolates From a Chinese Tertiary Care Teaching Hospital
Source: Front Microbiol. 2021 Aug 3;12:671153. doi: 10.3389/fmicb.2021.671153 (PMC8369762; doi:10.3389/fmicb.2021.671153)
Supplement: Supplementary file 2 [file Table_2.DOCX]

Table S2 The inhibition zone diameters of parental strains and respective resistant subpopulations

| Strains | Diameter of inhibition zone(mm)^a^ | |
| --- | --- | --- |
|  | Parental strains | Subpopulations |
| K10 | 18 | 11 |
| K24 | 18 | 12 |
| K26 | 18 | 12 |
| K86 | 22 | 11 |
| K89 | 23 | 12 |
| K98 | 20 | 12 |
| K116 | 20 | 11 |
| K118 | 20 | 12 |
| K130 | 19 | 12 |
| K148 | 21 | 12 |
| K151 | 18 | 12 |
| K182 | 21 | 12 |
| K191 | 19 | 12 |
| K194 | 20 | 12 |
| K197 | 20 | 12 |
| K228 | 18 | 12 |
| K289 | 18 | 12 |
| K295 | 18 | 12 |
| K300 | 20 | 12 |
| K320 | 18 | 12 |
| K326 | 19 | 12 |

^a^ The breakpoint of tigecycline is based on the EUCAST interpretive criteria, ≥16 mm as susceptible, ≤12 mm as resistant.
